# How people living with diabetes in Indonesia learn about their disease: A grounded theory study

**DOI:** 10.1371/journal.pone.0212019

**Published:** 2019-02-22

**Authors:** Titan Ligita, Kristin Wicking, Karen Francis, Nichole Harvey, Intansari Nurjannah

**Affiliations:** 1 Nursing, Midwifery and Nutrition, College of Healthcare Sciences, James Cook University, Townsville, Australia; 2 School of Nursing, Universitas Tanjungpura, Pontianak, Indonesia; 3 School of Health Sciences, College of Health and Medicine, University of Tasmania, Launceston, Australia; 4 Medicine, College of Medicine and Dentistry, James Cook University, Townsville, Australia; 5 School of Nursing, Universitas Gadjahmada, Yogyakarta, Indonesia; University of Wisconsin Madison School of Pharmacy, UNITED STATES

## Abstract

**Background:**

Diabetes education has been found to impact positively on self-management by people with diabetes although little is known about the process by which they assimilate information. The aim of this study was to generate a theory explaining the process by which people with diabetes learn about their disease in Indonesia.

**Methods:**

This study employed a grounded theory methodology influenced by constructivism and symbolic interactionism. A total of twenty-eight face-to-face or telephone interviews with participants from Indonesia that included people with diabetes, healthcare professionals, health service providers and families of people with diabetes were conducted in both Indonesia and Australia.

**Results:**

This study discloses a core category of *Learning*, *choosing*, *and acting*: *self-management of diabetes in Indonesia* as the basic social process of how people learn about their diabetes. The process includes five distinctive major categories. People with diabetes acted after they had received recommendations that they considered to be trustworthy. Factors that influenced their choice of recommendations to adopt are also identified.

**Conclusions:**

Awareness of the complexity involved in their decision making will assist healthcare professionals to engage effectively with people living with diabetes.

## Introduction

Abundant studies have shown that health education is critical for people living with diabetes, as the information provided assists them to self-manage their condition, maintain their blood glucose level (BGL) within a healthy range and prevent deterioration [1,2]. Health education improves people’s health literacy [3]. Health literacy refers to people using their skills, knowledge, abilities and experiences to seek, understand and take action on health information and consequently make a decision about health care as well as find and use health services [4]. Therefore, education allows people to become empowered and autonomous when making important decisions regarding their health [5]. When people engage in educational programs there is a positive link to better health outcomes for that person, which enhances their overall health and wellbeing [6–9]. Conversely, people that do not engage in health educational programs are more likely to make poor health decisions and engage in harmful self-management practices.

Educational programs provide information to highlight the importance of treatments to effectively manage diabetes and prevent complications, with the aim of promoting self-care management [7,10]. Educational guidelines assist people with diabetes to understand the rationales for recommended interventions, such as: regular BGL monitoring; administration of insulin and oral hypoglycaemic medications; low sugar diet; and lifestyle changes [1].

Unfortunately, participation in diabetes educational programs is limited [7] because of financial, medical and logistical (access) reasons [11]. This paper will explicate the process of how people with diabetes in Indonesia learn about their disease, which is crucial in trying to help improve the health outcomes for Indonesian peoples. Furthermore, an exploration of the experiences of people with diabetes will be shared so that the process of how they receive and then engage with education can be better understood, with the aim of providing more effective diabetes educational programs in the future.

## Background

Diabetes in Indonesia is considered a major health problem and has been a concern since the early 1980s [12]. With more than 10 million people living with diabetes, Indonesia has a prevalence rate of 6.2% [13] and diabetes is one major cause of death [14]. Indonesia was rated as one of the top ten countries globally with a high number of individuals living with diabetes in 2013 [15]. It is predicted that the same pattern will continue [15] unless interventions to prevent and manage diabetes are implemented.

To address the alarming numbers of Indonesian people with diabetes, diabetes experts have developed guidelines for preventing and managing diabetes [16]. Managing diabetes is crucial to prevent people from experiencing severe complications such as neuropathy, nephropathy, retinopathy, microvascular and cardiovascular disease [17–20]. Pharmacologic therapies such as oral medications and insulin, and non-pharmacologic therapies such as lifestyle modifications are still considered contemporary interventions for managing diabetes [21]. These interventions can only be implemented and achieved through diabetic educational programs that promote self-care management [22].

Healthcare services in Indonesia provide some effective diabetes educational programs [6,8,23]. The availability of certified diabetic educators is limited in Indonesia [24] resulting in a dearth of skilled health care professionals (HCPs) to provide education to people with diabetes [25,26]. There is little evidence available about the process of how people with diabetes learn about their disease after they have received diabetes related information informally or formally through structured educational interventions in the Indonesian context. We used a grounded theory methodology to gain insight into how people with diabetes learn about their disease and the processes by which they received and engaged in diabetes health education. Understanding this learning process has the potential to assist HCPs to develop and make available effective, creative and culturally-responsive diabetes health education to inform people with diabetes in their selection of appropriate self-management strategies.

## Grounded theory study

Ethical approvals to conduct the study were obtained from the Human Research Ethics Committee at James Cook University and from the local Research Ethics Board at the Faculty of Nursing, Universitas Indonesia. The research study avoided coercion because only participants who volunteered to participate in the study were interviewed. Before the interviews were performed, we provided the participants with information sheets and written consent forms. The interviews started once the participants had read and signed the informed consent forms.

### Methodology

This study employed a grounded theory methodology. Originally established by two scholars Barney Glaser and Anselm Strauss, grounded theory is a qualitative research methodology aiming to generate a theory grounded from the data [27]. The generated theory then explains the study phenomena. Two philosophical stances influenced this grounded theory study. Firstly, symbolic interactionism whereby people’s lives and behaviours were explored [28] and secondly constructivism, the process of how people understood their diabetes (meanings) and how that understanding subsequently informed their actions [29]. In this grounded theory study, we generated and concurrently analysed data. To rigorously employ grounded theory methodology, we used the essential methods of grounded theory in the selection of study participants (purposeful and theoretical sampling) and in the collection and analysis of data [30]. When using a purposeful sampling method, potential participants are targeted who are considered suitable to provide insights that will allow exploration of the phenomenon of interest [31]. However, theoretical sampling is unique to grounded theory, and is a method in which more data are gathered to define and refine the relevant developing categories or emerging theory [32].

The data were analysed by using constant comparative analysis through three stages of the coding process: initial coding, intermediate coding and advanced coding. Data were managed and organised through the NVivo software version 10 [33]. The process of analysis occurred through constant comparative analysis, which is referred to as comparing the data, specifically, comparing incidents with incidents, incidents with codes, codes with codes, codes with categories and categories with categories [30]. The comparisons were also applied between existing data and incoming data. For example, in the incoming data, new codes were developed. Then these new codes were compared with the formerly developed codes (existing data). Also, codes developed in one interview excerpt were compared with codes developed in other excerpts of the same interview, or codes developed in other interview excerpts.

Coding is defined when the analyst applies labels to data fragments and as a result the data has a connection to theory development [29]. In the initial coding stage, raw data was broken down into fragments and labels were attached to the data. This process produced codes. A process code was labelled when coding conceptual actions or observable activities and they are written by using the gerund form or ‘ing’ words, for example ‘seeking’ [29,34]. Initial coding took place both during the first and second data generation field trips.

Codes developed during initial coding were collapsed in intermediate coding, which is sometimes referred to as focused coding. At this stage, significant codes were selected to form categories [32]. Categories were then developed from these codes while continuing to apply constant comparative analysis. During the next stage of analysis, advanced coding, one core category was selected and it comprised a theory entitled *Learning*, *choosing*, *and acting*: *self-management of diabetes in Indonesia*. This core category has five major categories that elucidate the process of how people with diabetes learn about their disease.

Additionally, essential research aids such as: memo writing; concept mapping; field notes; and storyline were also employed to assist with analysis and theoretical integration. This study employed concept mapping as an analytical tool to explore the data and to link codes, categories and sub-categories. A storyline was crafted, as a tool for explaining the theory and was regularly updated during data analysis. Both the concept map and the storyline explain the theory entitled *Learning*, *choosing*, *and acting*: *self-management of diabetes in Indonesia*.

### Participants and study settings

The study was conducted in West Kalimantan, which is one of the 34 provinces in Indonesia and inhabited by three major ethnic groups. West Kalimantan has a high prevalence rate of diabetes. It was noted by the Indonesian Basic Health Survey that the prevalence rate of diabetes mellitus in that province rose from 0.8% in 2007 to 1% in 2013 [35,36]. A total number of 28 interviews were undertaken with participants from both inpatient and outpatient settings in and around Pontianak, the capital city of West Kalimantan province.

### Study design

This three phase study was undertaken between April 2016 and July 2017. All interviews were carried out in the national Indonesian language. The Indonesian language was also used during the three essential coding processes: initial; intermediate and advanced coding. Hence, a bilingual advisor was included on the research team to assist with the process of translating the interviews and analysing the data. For anonymity and confidentiality, pseudonyms are used when reporting participants’ quotes.

#### Phase one

Purposive sampling was used to select the participants in phase one and a scoping review also helped to inform which HCPs should be involved [25]. A scoping review is a framework that systematically reviews and scopes the available qualitative and quantitative literature to answer the questions and identify the nature of evidence in research studies [37–39]. Participants consisted of a person with diabetes, a nurse academic, outpatient clinic nurses, a pharmacist, a dietician and a specialist doctor. They were recruited from two general hospitals, a public health centre (*Puskesmas*) and a nursing academic institution in Indonesia. Each participant participated in a face-to-face interview, which was digitally recorded and conducted by the lead researcher in West Kalimantan. In this phase, concept mapping was employed.

#### Phase two

Data from the first phase led to further data gathering. As the analysis proceeded, theoretical sampling was used to select the next data that included a wide range of participants and additional research settings. Ethics amendment approval was obtained and a wider range of participants were recruited from additional settings, such as a private wound care clinic and two other public health centres. Interviews with a further 17 participants were again performed in person in Indonesia. They included eight people with diabetes, a general practitioner, a ward nurse, a nursing student, three family members, a health promotion staff member, an exercise instructor and a *kader* (an Indonesian term for a lay health worker). In this phase, an initial version of the storyline was crafted.

#### Phase three

Theoretical sampling directed phase three. The aim of this phase was to refine the storyline in order to facilitate the theory integration process. This process was to determine if the theory was suitable for the area from where it was generated and where it will be used [40]. Two participants were re-interviewed who were involved in the previous phases; an exercise instructor living with diabetes and a nurse academic. The two new participants were recruited and interviewed; a clinic nurse and a person with diabetes. All interviews were conducted via telephone from Australia to Indonesia with each of the 4 participants. Before the interviews, participants were provided with a storyline and a concept map written in the Indonesian language.

## Results

### Learning, choosing, and acting: Self-management of diabetes in Indonesia

**Fig 1:**
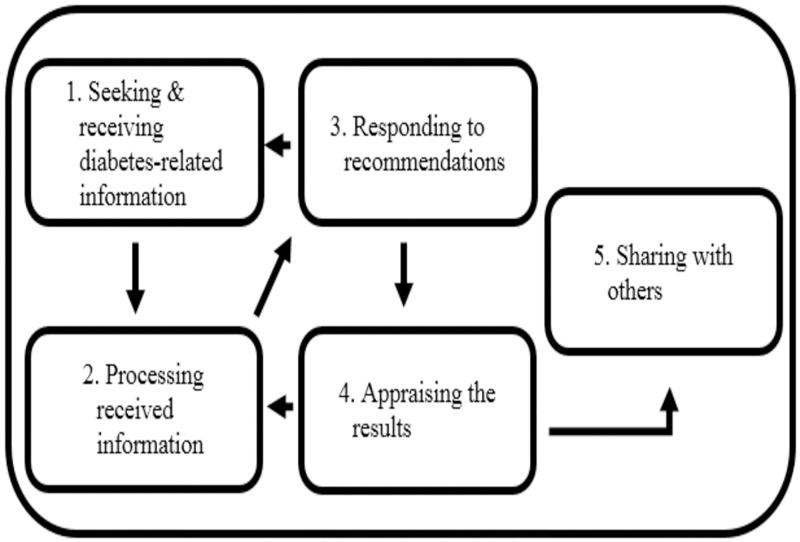
A process of Learning, choosing, and acting: Self-management of diabetes in Indonesia.

*Learning*, *choosing*, *and acting*: *self-management of diabetes in Indonesia* is the core category of this study that explicates the process of how people in Indonesia with diabetes learn about their disease. The process consists of five categories, which are the stages that people with diabetes navigate when learning about their disease: ‘seeking and receiving diabetes related information’; ‘processing received information’; ‘responding to recommendations’; ‘appraising the results’; and ‘sharing with others’. The process is displayed in Fig 1.

This diagram represents a theory of *Learning*, *choosing*, *and acting*: *self-management of diabetes in Indonesia*, a basic social process of how people with diabetes in Indonesia learn about their disease. The numbers show the categories of the process that occur in chronological order. This process occurs dynamically, both in linear and cyclical movements.

#### Category 1: Seeking and receiving diabetes-related information

This category can occur before and after people have been diagnosed with diabetes. Before the diagnosis, people with diabetes noticed or recognised their symptoms and gathered information about their disease through active and passive learning techniques. Active learning means that people asked questions of others or sought information from sources such as the internet, television or written materials. Once they were diagnosed, they actively sought information about the management of the disease, clarification about therapies being used, and explanations about why they were experiencing bodily changes related to the medication used. One example of this was Widya, an exercise instructor who has diabetes:

I asked questions about what diabetes is. … One [recommendation] that I got is to do exercise. I asked my friend who was in the nutrition division. She said, ‘”Do some exercise, try to do exercise”.

(Widya: G2P14)

Passive learning refers to receiving and listening to information from other people, commonly HCPs, without requesting the information themselves. This situation usually occurred when people with diabetes visited healthcare facilities such as an outpatient clinic or during hospitalisation. Information was then offered as part of the clinic consultation or in the case of hospitalisation, by HCPs caring for the person. For example, a pharmacist reflected on the explanations he provided:

…to [make people with diabetes] understand about the effect of taking medicine regularly and irregularly. We provide the explanation as such. In educating [them], we provide information in order to prevent complications from diabetes

(Cahyo: G1P10).

The source of the information (i.e. who delivers the information) and the place the information was given, varied. Sources of information, from HCPs, included: doctors (e.g. general practitioners and medical specialist doctors); nurses (eg. ward nurses, outpatient care nurses); dieticians; and pharmacists. In addition, non-HCPs may also be sources of information: *kaders*; health promotion staff at hospitals; nursing students; family members; friends; people with diabetes; and/or individuals who have a relative living with diabetes. The places where information was received included: healthcare facilities such as hospitals and outpatient clinics; within their communities when interacting with others; and/or attending health seminars. Haris is an example of someone who sought information from a non-HCP, his little brother:

I was eating and drinking enough… [but] the body was limp, no energy. I just wanted to have a rest. … I asked my little brother. He has diabetes before me. So I asked my brother why I have this unwell condition. He brought me to the medical [clinic]. I was checked up… everything was checked up. My blood sugar level was 488 [mg/dL]…very high. My little brother advised me. So, from there I started to learn how…yes…I take care of it [eating or drinking] until now.

(Haris: G2P1)

#### Category 2: Processing received information

The second category is processing the information received. Before the person could trust the information, they examined the information based on their prior knowledge, own experiences and personal judgement, as well as asking for a second opinion from who or what they believed to be a reliable source. Next, they had a choice, to trust or distrust the information. The distrusted information was dismissed while the trusted information was accepted. Larisa, a person living with diabetes, did not trust information given to her by friends and she decided to dismiss it:

They [friends] usually take alternative therapies. I do not trust the alternative therapies. I do not know the measurement, the amount, [and] the dosage. If we have over dosage, we’ll have difficulties. … So when my friends said “take this…take that”. I just said “yes…yes…”, but I did not take it. Unless it is authentic and there is a study/research on it, then I can understand it. If not, I do not trust it. I do not easily trust something

(Larisa: G2P7).

Zeta, another person with diabetes, also sought additional information to clarify her understanding of what medication could be used to manage her diabetes:

I received other people’s opinion and advice or friends’ advice. … I do not just directly execute any received advice. No. I have to look at Google, [for instance, about] the function of leaves A or leaves B. The side effect of them.

(Zeta: G3P1)

#### Category 3: Responding to recommendations

**Fig 2:**
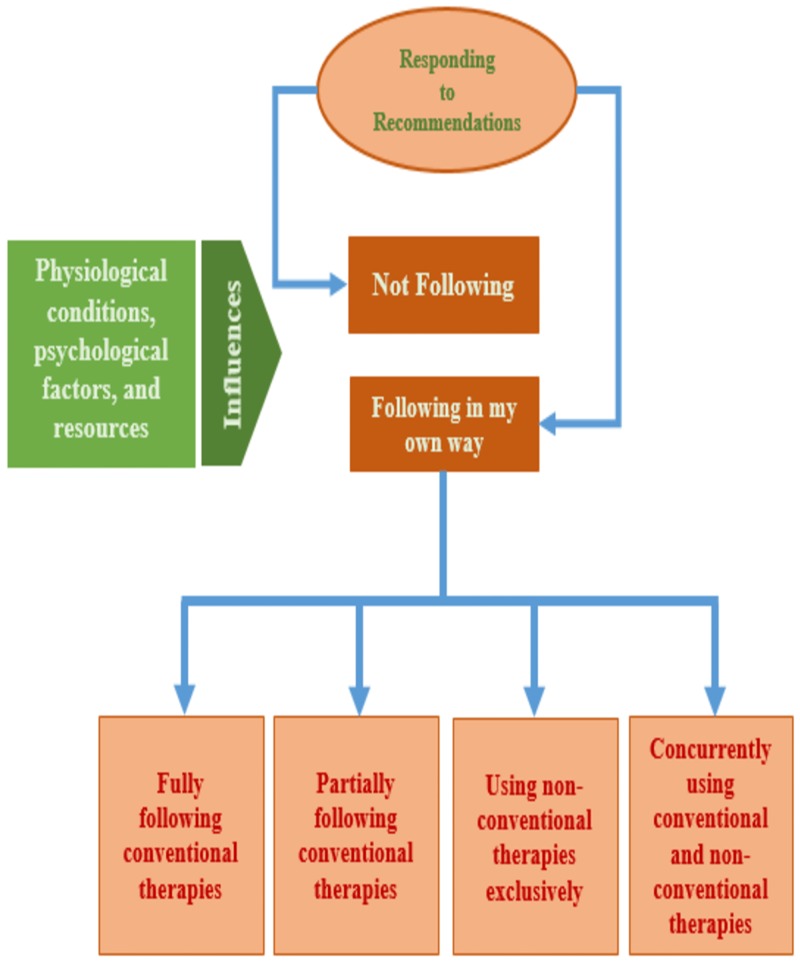
Responding to recommendations.

Individuals responded in either of two ways; they followed the recommendations suggested by either HCPs and/or non-HCPs or they did not follow recommendations from the HCPs or non-HCPs. The HCPs recommended conventional therapeutic interventions while non-HCPs suggested both conventional and non-conventional therapeutic interventions. There were a number of influencing factors that affected a person’s decision to follow or not to follow recommendations. These were: financial situation; time; geographical location; recommendations from relatives and friends; physiological reasons such as changes in the body or worsening symptoms; psychological reasons such as fear of side effects from the medication, fear of having to inject medication and underestimating the disease severity; and issues of convenience and practicality (See Fig 2).

This figure shows how people with diabetes responded to recommendations by either following or not following the recommendations. Their decisions were influenced by three factors: physical, psychological and availability of resources. People with diabetes selected one of four variations of responding to the recommendations.

Utama, a diabetic of 8 years, offered that he was influenced by his older sister who told him he would become dependent on insulin, so then he did not follow the HCPs’ recommendations:

I am afraid of becoming dependent on the medication, my sister said ‘don’t you use that insulin. If you use once, it will be forever’. My sister continued to say that right up until her death [from diabetes complications].

(Utama: G2P5)

The above quote shows that Utama followed his (older) sister’s recommendation rather than the recommendations from his HCP. He respected his sister’s experiential insight gained from living with the disease, but he also demonstrated respect for someone older than himself, which is a norm in Indonesian culture [41]. Utama was afraid of taking his prescribed insulin for two reasons: the above fear of becoming dependent as per his older sister’s recommendation, and an additional fear of injecting insulin:

I have been prescribed insulin but I don’t use it. I have never [used it]. I have been prescribed [insulin] when being discharged from the hospital. … I am afraid to inject it [the needle].

(Utama: G2P5)

Betty, a diabetic of 13 years, delayed commencing insulin injections for a year because of cost, inconvenience and fear of having to give herself an injection:

Firstly I didn’t want to. For a year I didn’t want to. I was really afraid of [injection]. Doctor B who keeps talking to me a lot. It has been a year [since] he told me to have an insulin injection. … I did not want to because insulin is troublesome. I have to check my blood sugar by myself. Then if [the blood sugar] decreases [too much], [I will have] faintness, [and] I cannot go anywhere. I thought for a year. I did not want. But [I was] getting thinner.

(Betty: G2P2,3,5)

Another reason to delay treatment was when people with diabetes underestimated the severity of the disease when comparing their experience of diabetes with other people’s. They often perceived that their diabetes was ‘not too bad’ compared to other people whose diabetes they believed was more severe. Utama explained his diabetes was not as significant compared to his sister who had died as a result of a diabetic foot ulcer:

…in the last four years, I just combat [the disease]. People say that we don’t need to…worry about this disease. If I think too much, it will be harder so I don’t think about it too much. Now, I think my disease is not too severe. I haven’t got the one that my sister had. My sister had a hole [diabetic foot ulcer]… I am not that severe. I only have these boils. It hasn’t been severe. Not like other people who have a small wound then it gets swollen…. No, I am not like that [not that severe].

(Utama: G2P2)

Ranti, a diabetic of four years, was concerned about the drug’s side effects on her body:

She [the doctor] gave me a medication…. I do not take doctor’s medication [anymore]… [because of] the chemical [materials]. I took the medication from the doctor only for one week.

(Ranti: G2P1)

In addition to individuals who chose not to follow recommendations, there were individuals who only followed recommendations that they trusted, but did so with varying levels of commitment. Under the umbrella of following recommendations in their own way (see Fig 2), they either fully followed conventional therapeutic interventions; partially followed conventional therapeutic interventions; used non-conventional therapeutic interventions exclusively; and/or followed conventional and non-conventional therapeutic interventions concurrently.

People who fully followed conventional therapeutic interventions reported adherence to medication(s), exercise, diet and BGL monitoring. People who partially followed conventional therapeutic interventions tended to adhere to one or two interventions only. Kevin, a person with diabetes, stated he only took the medication.

I cannot restrict my diet. I still have appetite to eat. [I do] regularly [take my medication]. … [I] never had [diabetes exercise]. It’s in PHC but I do not want to, [because] I am lazy to do [so].

(Kevin: G2P2,5)

People who used non-conventional therapies exclusively did not take prescribed medication of any kind or any other conventional therapeutic interventions. These people relied on non-conventional therapies such as herbal or traditional medication, factory-made herbal medication or a device worn on their body. One person wore a magnetic girdle, as she believed it to be beneficial in helping with her diabetes.

I wear this supporting device. [It is like a corset/waist belt]. For what I have known, it repairs …Diabetes occurs because our pancreas is damaged… The pancreas cells are damaged. Or kidneys [are damaged]… If we take many medications [and] drink less water, it can damage the kidneys. So, I am helped by buying this device so that my kidneys will be good. This device only contains [a] magnet.

(Ranti: G2P4)

The final subset of people used both conventional and non-conventional therapeutic interventions, or alternatively chose to *follow the recommendations*, but *in their own way*. This subset of people chose to trial both therapies either consecutively or concurrently for a short period of time. Kevin, a diabetic of five years, indicated that he sometimes took both conventional and non-conventional therapies concurrently:

I take prescribed medication by turns [intermittently], sometimes I also take herbals.

(Kevin: G2P1)

The phenomena of taking conventional and non-conventional therapies concurrently was a concern for HCPs, who feared that people may be at risk of hypoglycaemia. Aditya, a nurse academic and clinician, explained that he occasionally consulted with people with diabetes who took both therapies in the same day. He stated:

There were also some patients telling us “I have taken this [non-conventional therapy] but I took medication from the doctor concurrently. I separated it by approximately two hours”. After we tested the BGLs, it showed that the BGLs were stable. The most important thing that we educate about is the hypoglycaemia. “If you take the therapy and you have these symptoms [hypoglycaemia symptoms], it means that you have to stop the herbal”.

(Aditya: G3P2,3)

#### Category 4: Appraising the result

Regardless of whether the person followed or did not follow recommendations advocated by HCPs and non-HCPs, these people appraised the results to determine if what they were doing was making any difference to their health. There are three sub-categories of ‘appraising the result’: measurement, progress and further actions. People with diabetes observed their body changes either subjectively or objectively for positive and negative changes or for no changes. For instance, Oscar demonstrated appraising the result by objective measurement:

I tried [herbal therapy] from browsing the internet. It really decreased it [the BGL]. Then I became more motivated to take it [the therapy]. It decreased it [the BGL] again.

(Oscar: G2P3,4)

In the following quote, Zeta found desirable (positive) changes related to her diabetes condition:

Zeta: I was suggested to take herbal, a product from New Zealand. It’s a capsule… I was observed [by optometrist] regularly on my eyes. Alhamdulillah [thanks be to God] the [blurred eyes] are gone. I feel comfort in my body.

(Zeta: G3P3,4)

Whereas, Haris expressed that he had been taking traditional medication, but when he reflected on how it was working he found it had made no difference to his condition:

I firstly didn’t want to have chemical medication. By taking the traditional medication that I made by myself, I might find the solution, but in fact, there wasn’t. No change [progress].

(Haris: G2P5)

People in this category used their experience to decide whether they would continue with what they were doing or not, to manage their diabetes. Viola, a spouse of a person with diabetes recounted her decision to modify how they managed her husband’s diabetes:

We tried a herbal medication for several years. No progress. So, we decide to be managed by a doctor. To regularly go to doctor consultations so that he [the husband] can get better.

(Viola: G2P8)

The outcomes that people identified were based on their lived experiences. The category of *processing received information* refers to the ‘experiential insight’ that people developed, that in turn then helped them decide whether to continue or discontinue their current treatment and whether or not to return to category 1 to seek further information or recommendations, thus illustrating the cyclic nature of the process.

#### Category 5: Sharing with others

The last category is ‘sharing with others’. Sharing with others encompasses what information to share and with whom to share it. People tended to share their experiences with people they knew best, particularly family members such as children or spouses. Haris recounted:

I keep looking forward and prepare myself. I said to my wife and children. ‘I have this disease. This disease [can] affect anywhere [inside the body].’ I inform them so that they know. I am not frightening them. One day it will happen. With or without the disease, we will all die. But with [me having] this disease, I said, ‘you must be aware of… you must change your life concept. You must keep healthy. Don’t be careless’.

(Haris: G2P8,9)

**Fig 3:**
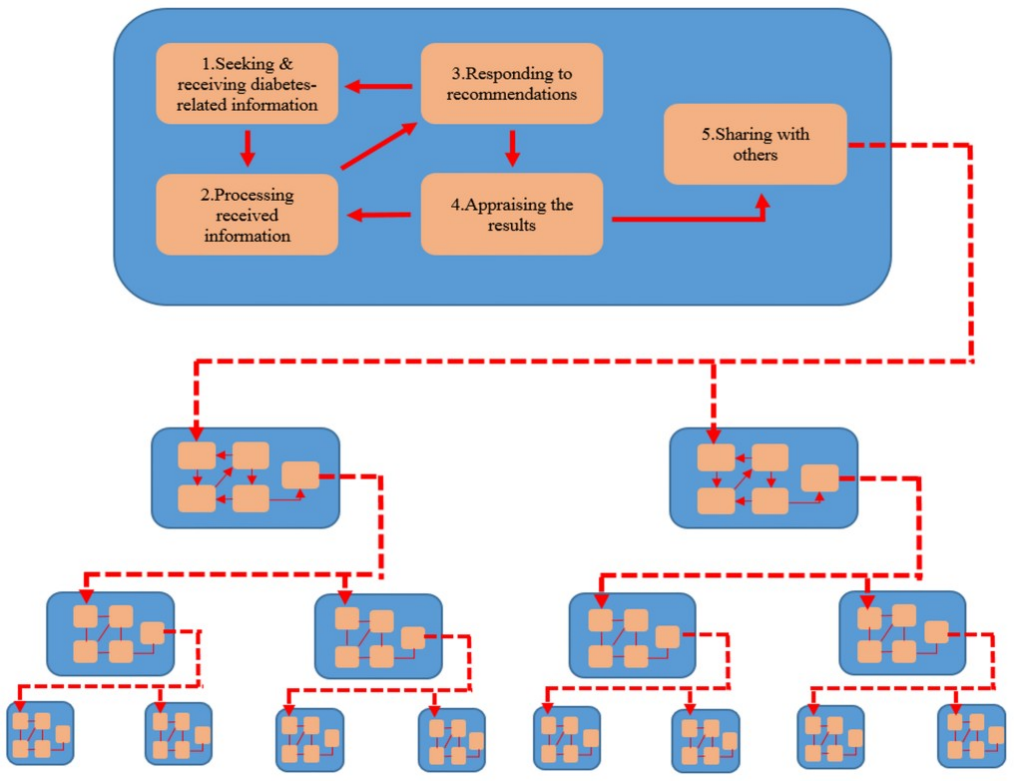
A sharing phenomenon among people with diabetes.

Through interactions in the community, people with diabetes shared their own insights with other diabetics and also with people in the community who may have a loved one with diabetes. When people with diabetes shared their experiences with another person, that conversation was often the beginning of that next person starting their own process of *Learning*, *choosing*, *and acting*: *self-management of diabetes in Indonesia*. Fig 3 displays the phenomenon of a person with diabetes sharing insight or experience with another person living with diabetes.

This figure shows how the process of *Learning*, *choosing*, *and acting*: *self-management of diabetes in Indonesia* occurs from one person with diabetes to another person with diabetes. The process starts when a person with diabetes shares their experience or information with another person with diabetes. The process of sharing information continues from one person, to the next, to the next, etc.

Utama shared his own experiences with other people he knows who are also living with diabetes:

I often inform them. It is about the symptoms. They do not know what diabetes looks like. I said, ‘if you want to prove whether you have diabetes or no, you collect your urine. If there are ants in there, you may have diabetes. The symptoms are dizzy, fatigue, weakness and thirsty. You just want to drink water or sweet drink.’ Those are the symptoms [that] I said to them.

(Utama: G2P10)

## Discussion

### Where is diabetes related information sourced from?

It is not uncommon for people with diabetes, especially those who experience severe symptoms, to seek information and recommendations from HCPs [42]. People with diabetes involved in our study also sought information from a variety of people other than HCPs, namely: friends, acquaintances and family members. This result is congruent with a study conducted in India, where people with diabetes learned about their disease through a variety of sources such as books, media and friends [43]. When a diversity of sources are used, there is a high risk for people to be exposed to less reliable or unreliable recommendations.

Recommendations from others may influence people’s decisions to stop, alter or to maintain their current diabetes care. In this present study, people sometimes discontinued practices after receiving information from non-HCPs. They also may have changed from using conventional therapies to non-conventional therapies, or vice versa. Hjelm and Atwine [42] reported that people seek non-conventional therapies, such as herbal medications or traditional healers, when they perceive that conventional therapy has failed them. This study found that receiving recommendations from HCPs does not preclude people from also seeking information elsewhere, similar to Mendenhall et al.’s [43] findings in India.

### Choosing information

People with diabetes selected information based on their prior knowledge, lived experience personal judgementand/or other people’s opinions. Additionally, the level of expertise of HCPs and the lived experience of other people with diabetes were also taken into account. People with diabetes trusted the opinions of other people living with diabetes and were sceptical of the recommendations of people who were not diabetic. Sometimes, people with diabetes accepted recommendations from their relatives, who were living with diabetes, rather than the recommendations of their HCPs. The recommendation from the HCPs that people with diabetes in this study chose not to follow usually centred around taking prescribed medications. Many of the study participants believed that the chemical substances in prescribed medications would cause more harm than good, thus they decided not to follow the HCP’s recommendation. In contrast, some participants who did follow the recommendation from their HCPs rather than from other people, did so because they acknowledged their HCP’s level of expertise. Therefore, they put more trust in their HCPs rather than in non-HCPs.

Building trust between people with diabetes and HCPs is crucial during the provision of care. A previous study did find that in general, satisfaction and adherence with diabetes treatment is associated with better communication between people with diabetes and their HCP [44]. However, this present study provides additional explication of that communication process, by showing how people with diabetes selectively choose which HCP information they will or will not trust and follow. White et al. [45] also found that a high level of mistrust can develop because of poor HCP communication skills and not including people in decision making about their own health. When HCPs do not involve people in their care, it may impair the development of a positive partnership between the person and their HCP.

### Following or not following recommended therapeutic interventions

Individuals in this study chose to adopt conventional and/or non-conventional therapies. This phenomenon is similar to that described by Mendenhall et al. [43], where some people reported using medical treatment (conventional) while others also reported using non-allopathic (non-conventional) therapies such as herbal medications, yoga and foods. However, the findings of this study are distinctive in showing that some people with diabetes elected to partially follow recommended conventional therapeutic regimes. Some people sometimes follow the recommendation in their own way by modifying the recommended therapeutic regime based on their own understanding. Their modifications could render the treatment ineffective or even harmful. This finding aligns with those of Webster et al. [46] who found people can develop questionable strategies to manage their diabetes when communication with their HCPs has failed. This phenomenon should be acknowledged by HCPs to ensure people with diabetes understand and are able to implement the therapeutic strategies proposed by the HCP rather than their interpretation or modification of that proposed strategy.

Resource issues, such as affordability and accessibility of therapies, were found to be factors influencing peoples’ decisions to follow recommendations in this present study. Others factors were physiological and psychological reasons. Even though people in this study could and did make their own decisions, they ideally should be guided by a HCP so that appropriate therapies are implemented. Therefore, our findings indicate that HCPs need to identify what challenges people with diabetes have encountered in following the recommendations from their HCPs and to then work together to see how those challenges can be resolved. Lee et al. [47] also found that during consultation, the HCP needs to consider each person’s knowledge level, personal traits, family supports as well as their awareness of diabetes medications and available treatment options. Our findings echo the Australian College of Nursing’s statement that involving the person in decision making demonstrates the HCP’s respect for the person and exemplifies a person-centred approach to care [48]. Our findings are congruent with those of Herlitz et al. [49] and Ramsay Wan, Vo and Barnes [50] who found that people who trust their HCPs and feel empowered to ask questions and make informed decisions are more likely to effectively manage their diabetes [49,50].

Respecting one’s cultural background should be acknowledged when HCPs work with people with diabetes. Due to cultural influences, some people with diabetes in this study still believed in and preferred the use of natural substances, either solely or in combination with prescription medicines, as their way of managing their diabetes. The use of natural substances has been practiced by Indonesian people for generations for disease treatment [51]. The use of natural substances for disease management may still be practiced by people who live in rural areas and have limited access to prescription medication [52]. The cultural context must be taken into account when working with Indonesian people with diabetes. It is recommended that HCPs provide information regarding the evidence for the effective use, dosage, and the route of administration of natural substances to people with diabetes who use these to manage their disease. Identifying culturally-influenced practices can assist HCPs to understand the rationale for people with diabetes choosing disease management methods. This information will ensure that HCPs are able to provide appropriate health education and support people’s diabetes management regimes.

### Evaluating the care

Experiential insight gained from this action is a distinctive feature of the theory of *Learning*, *choosing*, *and acting*: *self-management of diabetes in Indonesia*. Learning about diabetes led to people gaining insight in this present study. This finding reflects those of Quandt et al. [53] who claimed that what people learn influences their diabetes related beliefs and their capacity to manage their diabetes.

People in this study appraised their current therapy to decide whether to continue or discontinue their current regime. The literature highlights that people experience positive and negative outcomes following initiation of treatment [54,55]. This current study demonstrated that people with diabetes evaluated their treatment using objective and subjective measurements. They observed their body changes subjectively and objectively to determine desirable and undesirable outcomes. This appraisal action is part of their learning about their diabetes and its management, and resulted in their newfound experiential insight.

People relied on a subjective measurement, which was based on how their body felt, rather than objective biometric assessment. Purchasing a BGL monitor is an expense that many Indonesian people with diabetes were unable to afford. The cost of buying a BGL monitor would currently equate to 25% of the average monthly income of an Indonesian person [56]. Participants reported that access to services such as a community pharmacist that provides BGL monitoring was not always locally available. As a result, regular monitoring of their BGL was not possible.

People in this study compared their experience of diabetes to other people’s experience of diabetes. This comparison caused them to underestimate their own disease severity, since they had not yet experienced complications such as foot ulcers or diabetic retinopathy. This finding is confirmed by Pitaloka and Hsieh [57] who found that people with diabetes in Indonesia considered diabetes was not life threatening as they were able to perform every day tasks. Underestimation of the severity of diabetes by participants in our study led to them delaying implementation of treatment or to modifying their treatment plans. Participants who experienced secondary complications subsequently sought medical assistance, although for some people the extent of deterioration limited the therapeutic interventions that could then be implemented. For these people, death was inevitable.

People in the current study altered their diabetes management regimes if undesirable outcomes were experienced, new information regarding their current therapy was received and if they were unable to secure medications or necessary equipment such as insulin or BGL monitors. This study’s findings concur with that of Hjelm and Atwine [42] who identified that people may modify their therapeutic regimes when they are not satisfied with the outcome.

Other studies have found that experiences of implementing diabetes self-management can be exhausting and frustrating because people with diabetes have difficulties in balancing their everyday life especially when dealing with medication regimes, meal planning, compensating for a sedentary working life and avoiding self-care exhaustion [54,58,59]. Participants in the present study also reported similar challenges and frustrations. Therefore, it is recommended that HCPs engage early in a thorough, respectful and sensitive discussion with people with diabetes to discuss their understanding and management of diabetes and the difficulties they encounter in following treatment recommendations. These conversations should include provision of information to address knowledge deficits and adaptations if appropriate to treatment recommendations or assistance with addressing identified difficulties.

### Informing others about their experiences

A person often acquired information about diabetes from hearing about other people’s experiences with diabetes. Consequently, that person then went on to share his/her own experiences of diabetes with other people. In this study, the most likely people that a person shared their experience with were significant others, such as family or friends, and more rarely, acquaintances. People with diabetes will share information about their diabetes to others that they believe can provide feedback including HCPs [60].

People with diabetes can also learn from the stories of others who also have diabetes [61]. For instance, AlQarni, Yunus and Househ [62] found that sharing stories or experiences and information related to diabetes with each other and/or their caregivers occured via electronic mediums such as social media [62]. However, people in this current study still shared their experiences in person, during social interactions with families, friends, neighbours and community groups. In Indonesia, living in a neighbourhood allows people to gather and interact with each other on social occasions, either formally or informally. These occasions provide an opportunity for people with diabetes to discuss with others their experiences of living with diabetes. Consequently, people can both share their experiences and hear experiences from others about diabetes and how others manage it. The information shared may be appropriate or erroneous. Both social media and face to face social interaction can be useful mediums to share experiences. However, HCPs need to be aware that incorrect or misleading information may also be shared in these non-moderated contexts, as was also reported by Edwards et al. [63].

## Study limitations

Participation in the study was voluntary, and as in any qualitative study, the use of volunteers may introduce a volunteer bias. The results therefore may not be applicable to the entire population in the study setting. Additionally, the study participants came only from two of the three main ethnic groups in West Kalimantan, which may limit generalisability to all Indonesian people with diabetes.

## Conclusion

This study produced a theory entitled *Learning*, *choosing*, *and acting*: *self-management of diabetes in Indonesia*, which helps explain how people with diabetes learn about their disease and engage in health education in the Indonesian context. The process involves five categories which interact with each other in both a linear and cyclical fashion. The first of the five categories demonstrates how people with diabetes initially sought out information; often from family and friends who also had experiences with diabetes. Next the person proceeded to process this received information to make sense of it. From here people responded to the processed information by either following the recommendations or not. This study uncovered evidence of a number of factors that influenced whether a person followed or did not follow recommendations. Some of these factors were related to their financial situation, where they lived, what relatives and friends were advising and psychological reasons such as fear of having to inject medication. The fourth category involved people appraising their results and determining what future actions to take. The final category, sharing with others, was based on sharing information based on their own experiences.

Evidence from this study can inform HCPs to increase their awareness to focus not only on what people with diabetes have to do for managing their diabetes but also to evaluate how people with diabetes can do so, given the unique availability of resources they have, thus exemplifying person-centred diabetes care. The theory of *Learning*, *choosing*, *and acting*: *self-management of diabetes in Indonesia* can be a basis for HCPs to develop a feasible approach in health education that considers people’s own prior knowledge, personal judgement and own experience, as these factors can influence their decisions to employ appropriate diabetes self-management. Thus, to enhance monitoring and support of peoples’ self-management practices, HCPs need to have close and regular interactions with people with diabetes. Programs of diabetes specialisation in each discipline of health care should include the skills of close observation and detailed evaluation to ascertain how each individual with diabetes has arrived at their own current self-management approach, and how it is currently working for them (or not).

Further research is required to develop and evaluate a feasible model of diabetes care involving various related health disciplines in the provision of health education that exemplifies person centred care. Research could also investigate whether aspects of the theory of *Learning*, *choosing*, *and acting*: *self-management of diabetes in Indonesia* can be applied to other chronic diseases or to people with diabetes in other similar geographical locations.
